# JiangyaTongluo decoction ameliorates tubulointerstitial fibrosis via regulating the SIRT1/PGC-1α/mitophagy axis in hypertensive nephropathy

**DOI:** 10.3389/fphar.2024.1491315

**Published:** 2024-12-12

**Authors:** Yun Zhao, Qi Jia, Gaimei Hao, Lin Han, Yushan Gao, Xiaoyu Zhang, Ziming Yan, Boyang Li, Yiping Wu, Boya Zhang, Yubo Li, Jianguo Qin

**Affiliations:** ^1^ Dongfang Hospital, Beijing University of Chinese Medicine, Beijing, China; ^2^ Dongzhimen Hospital, Beijing University of Chinese Medicine, Beijing, China; ^3^ Institute of Basic Theory for Traditional Chinese Medicine, China Academy of Chinese Medical Sciences, Beijing, China; ^4^ School of Basic Medicine, Beijing University of Chinese Medicine, Beijing, China

**Keywords:** hypertensive nephropathy, tubulointerstitial fibrosis, mitochondrial dysfunction, SIRT1, mitophagy, Chinese medicine

## Abstract

**Introduction:**

With the increasing prevalence of hypertension, the incidence of kidney diseases is also increasing, resulting in a serious public burden. Jiangya Tongluo decoction (JYTL), a recognized prescription in traditional Chinese medicine (TCM), is commonly used to calm an overactive liver and reduce excess yang, while also promoting blood flow to alleviate obstructions in the meridians. Previous research has indicated that JYTL may help mitigate kidney damage caused by hypertension; however, the underlying mechanisms have not been thoroughly assessed.

**Methods:**

First, an amalgamation of UPLC-QE/MS and network pharmacology techniques was employed to pinpoint potential active components, primary targets, and crucial action mechanisms of JYTL in treating hypertensive nephropathy (HN). Then, we used spontaneous hypertensive rats (SHRs) and Wistar-Kyoto rats (WKYs) to evaluate the efficacy of JYTL on HN with valsartan as a positive reference. We also conducted DCFH-DA fluorescence staining in rat renal tissues to detect the level of ROS. Western blotting and immunohistochemistry were performed to investigate further the effect of JYTL decoction on key targets and signaling pathways.

**Results:**

Through UPLC-QE/MS and network analysis, 189 active ingredients and 5 hub targets were identified from JYTL. GSEA in the MitoCarta3.0 database and PPI network analysis revealed that JYTL predominantly engages in the Sirt1-mitophagy signaling pathway. Tanshinone iia, quercetin, and adenosine in JYTL are the main active ingredients for treating HN. *In vivo* validation showed that JYTL decoction could improve kidney function, ameliorate tubulointerstitial fibrosis (TIF), and improve mitochondrial function by inhibiting ROS production and regulating mitochondrial dynamics in SHRs. JYTL treatment could also increase the expression of SIRT1, PGC-1α, Nrf1, and TFAM, and activate PINK1/Parkin-mediated mitophagy.

**Conclusion:**

JYTL decoction may exert renal function protective and anti-fibrosis effects in HN by ameliorating mitochondrial function and regulating the SIRT1/PGC-1α-mitophagy pathway.

## Graphical Abstract

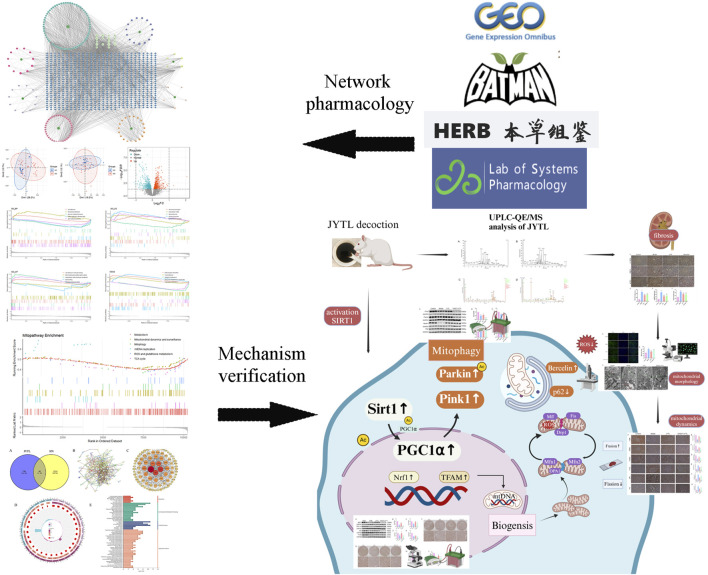

## Highlights

• JYTL has significant potential as a therapeutic intervention for hypertensive nephropathy.
• JYTL improved mitochondrial structure and repaired mitochondrial dynamics in SHRs.
• JYTL decoction enhanced mitochondrial function by promoting mitochondrial biogenesis
• JYTL decoction might relieve hypertensive nephropathy by regulating the SIRT1/PGC-1α/mitophagy signal pathway.

## 1 Introduction

Hypertension, one of the world’s most common chronic non-communicable diseases, is a leading cause of stroke, vascular disease, myocardial dysfunction, and kidney injury (Ko et al., 2023). Hypertension and kidney diseases are closely interconnected. Data from the United States Renal Data System revealed that the incidence of hypertension-induced kidney disease ranked second to diabetic end-stage renal disease (ESRD) (Weldegiorgis and Woodward, 2020). Renal damage from hypertension is progressive and the early symptoms are often insidious. Uncontrolled high blood pressure commonly leads to pathological changes in the kidney, including inflammatory infiltration, podocyte depletion, and hyaline degeneration of arterioles, leading to renal tubule atrophy, glomerulosclerosis, and TIF (Ni et al., 2024). Studies have shown that compared with glomerulosclerosis, TIF is considered to be a crucial determinant leading to ESRD (Wang et al., 2024; Rende et al., 2023; Liu and Zhuang, 2019). Hence, early intervention has been of great importance in delaying the progression of TIF and reducing the incidence and mortality of ERSD.

Emerging evidence has proven that mitochondria play a role in TIF (Zhang L. et al., 2023). Mitochondrial injury, characterized by the mitochondrial genome released from dying cells, is considered a biomarker to better identify chronic renal injury in hypertensive patients (Eirin et al., 2016). Under the pathological conditions of ischemia and hypoxia in hypertensive kidney damage, the relative excessive accumulation of reactive oxygen species (ROS) could break cellular homeostasis, resulting in oxidative stress and mitochondrial dysfunction (Irazabal and Torres, 2020). This triggers the activation of transforming growth factor (TGF) β, and other signaling pathways, releasing inflammatory and fibrogenic factors that contribute to TIF (Zhang et al., 2024). Thus, clearance of damaged mitochondria is critical for cell survival to reduce the concentration of ROS (Tang et al., 2021). As an adaptive or defensive mechanism, mitophagy contributes to selectively eliminating damaged or dysfunctional mitochondria. In mammalian cells, the Serine/threonine-protein kinase PINK1 (PINK1) and the E3 ubiquitin-protein ligase parkin (parkin) act cooperatively in sensing mitochondrial functional state and marking damaged mitochondria for disposal via the autophagy pathway to maintain mitochondrial quantity and quality in a variety of cell types (Eiyama and Okamoto, 2015). Recent observations have reported that PINK1/Parkin-mediated mitophagy can significantly improve mitochondrial function in renal tubule cells to alleviate TIF in kidney disease (Sang et al., 2020; Han et al., 2021; Chen H. et al., 2024). Therefore, Searching for therapeutic strategies to improve mitophagy appears to be a novel treatment option for TIF.

NAD-dependent protein deacetylase sirtuin-1 (SIRT1) is a highly conserved member of the family of NAD^+^-dependent Sir2 histone deacetylases, which deacetylates downstream Peroxisome proliferator-activated receptor gamma coactivator 1-alpha (PGC-1α) and consequently increases its activity (Tang et al., 2017). PGC-1α, a master regulator of mitochondrial biogenesis, which generates fresh, functional ones is essential for maintaining mitochondrial function and antioxidant capacity (Fontecha-Barriuso et al., 2020). Accelerating mitophagy during mitochondrial biogenesis may prevent over‐burdening or over‐crowding of the cell with excessive mitochondria, thus maintaining normal mitochondrial and organismal physiology. Studies have shown that the activation of the SIRT1/PGC-1α pathway can increase the level of mitophagy (Liang et al., 2020; Han et al., 2023). Additionally, mitochondrial DNA (mtDNA) stability, crucial for mitochondrial function and ROS elimination, is largely governed by the mitochondrial transcription factor A (TFAM), which is regulated by PGC-1α (Zhang M. et al., 2023), further highlighting the significance of the Sirt1-PGC-1α-TFAM pathway in mitigating TIF.

As a supplementary treatment, Traditional Chinese medicine (TCM) has been widely approved in East Asia for the treatment of hypertension and its related symptoms. Increasing evidence from systematic reviews and randomized controlled trials (RCTs) with rigorous methodological quality suggest that TCM could contribute to lowering blood pressure and relieving hypertension-induced renal injury (Lai et al., 2022; Xiong et al., 2015; Ya-Wei et al., 2020; Zhang et al., 2020). Jiangya Tongluo (JYTL) decoction is constituted by *Nacre, Senna obtusifolia (L.) H.S.Irwin & Barneby, Chrysanthemum × morifolium (Ramat.) Hemsl., Scutellaria baicalensis Georgi, Salvia miltiorrhiza Bunge, Achyranthes bidentata Blume, Carthamus tinctorius L.* and *Spatholobus suberectus Dunn.* The plant name was checked using “World Flora Online” (www.worldfloraonline.org). All eight herbs in JYTL decoction have been traditionally used to treat hypertensive nephropathy (HN), and multiple studies have reported the combined use of pairs of these eight herbs. For example, according to the prescription rules of famous old TCM experts for treating hypertension, Chrysanthemum morifolium Ramat., Scutellaria baicalensis Georgi., Salvia miltiorrhiza Bge., Achyranthes bidentata Blume, Carthamus tinctorius L. (Zou et al., 2007); the herb pairs Salvia miltiorrhiza Bge. and Carthamus tinctorius L. were identified as highly co-occurring herbal pairs, which reflected the important herb combinations of TCM against hypertension (Yang et al., 2024). Moreover, the component of JYTL ameliorated Renal Fibrosis by regulating mitochondrial dysfunction induced by oxidative stress in unilateral ureteral obstruction rats (Jia et al., 2021). Our previous studies have shown the prescription has protective effects on renal functions, decreasing 24-h urine protein, serum creatinine (Scr), and blood urea nitrogen (BUN) in SHRs (Han et al., 2015a). However, the mechanisms of JYTL in the treatment of hypertensive nephropathy need further investigation.

Hence, in this study, we focused on the effects of JYTL on renal interstitial fibrosis and mitochondrial function. We found that JYTL ameliorated mitochondrial dysfunction, reduced ROS generation, promoted mitochondrial biogenesis, and regulated Pink1/Parkin-mediated mitophagy in SHRs with renal damage. Our findings provide better insight into the molecular mechanism of JYTL as a treatment for HN.

## 2 Materials and methods

### 2.1 Animals and drugs

In this investigation, 10 Wistar-Kyoto rats (WKYs, 14–16 weeks of age, 220 ± 20 g) and 40 male spontaneously hypertensive rats (SHRs, 14–16 weeks of age, 220 ± 20 g) were collected from Beijing Vital River Laboratory Animal Technology Co., Ltd. (Beijing, China, License No. SCXK Jing 2021-0006). The animal operation in this study was carried out following the “Guiding Principles in the Use and Care of Animals” published by the US National Institutes of Health (NIH Publishing, No. 85–23, revised in 1996). This procedure was completed under the supervision of the Laboratory Animal Ethics Committee of the Institute of Basic Theory for Chinese Medicine, China Academy of Chinese Medicine Science. (IACUC Issue NO. IBTCMCACMS21-2303-02). All animals were kept in a clean room at 22°C ± 2°C and had free access to water and food.

JiangyaTongluo decoction is composed of eight individual traditional Chinese medicinal materials. The names and dosages of materials are shown in Table 1. All materials were extracted, concentrated, dried, and processed into granules. The granules of each material could be purchased in Dongfang Hospital affiliated with Beijing University of Chinese Medicine. The granules used in the current study were provided by Sichuan Neo-Green Pharmaceutical Technology Development Co., Ltd., (Sichuan, China). Valsartan was purchased from Novartis Pharma Ltd., (Beijing, China) and the Selisistat (Ex527, HY-15452) was purchased from MedChemExpress (New Jersey, United States).

**TABLE 1:** Composition and doses of JiangyaTongluo decoction.

Botanical plant name	Chinese name	Herbal part of use	Doses (g)	Composition (%)
Nacre	Zhen Zhu Mu	Seashell	30	20.4
Cassia obtusifolia L.	Jue Ming Zi	Seed	15	10.2
Chrysanthemum morifolium Ramat.	Ju Hua	Flower	15	10.2
Scutellaria baicalensis Georgi.	Huang Qin	Radix	12	8.2
Salvia miltiorrhiza Bge.	Dan Shen	Radix and rhizome	25	17.0
Achyranthes bidentata Blume	Niu Xi	Radix	15	10.2
Carthamus tinctorius L.	Hong Hua	Flower	10	6.8
Spatholobus suberectus Dunn	Ji Xue Teng	Caulis	25	17.0

### 2.2 UPLC-QE/MS analysis

The compounds in JYTL decoction were identified using a UHPLC System (Dionex Ultimate 3,000, Thermo Corporation, United States) coupled with Q Exactive high-resolution mass spectrometer (Thermo Fisher Scientific; Shanghai, China). Separation was performed on Waters UPLC HSS T3 column (1.8 μm particle size, 2.1 mm × 100 mm dimensions) at 40°C. The mobile phase consisted of solvent A (water with 0.1% formic acid) and solvent B (acetonitrile). The gradient elution conditions were as follows: 0–1 min, 98%A + 2%B; 1–41 min, 100% B; 41–50 min, 100% B; 50–50.1 min, 98%A + 2%B and 50.1–52.0 min, 98%A + 2%B. The flow rate was set at 0.3 mL/min, and the injection volume was 10.0 μL. A mass spectrometer equipped with an electrospray ionization source was used for both positive and negative ion modes with a mass range of 100–1,500 m/z. The ionization voltages were 3.7 kV (positive mode) and 3.5 kV (negative mode) and the capillary temperature was 320°C. Xcalibur 2.2 SP1.48 software was used for data collection and analysis.

### 2.3 Network pharmacology analysis of JYTL for HN treatment

#### 2.3.1 Screening of bioactive compounds in JYTL

First, the chemical components of JYTL decoction were retrieved from the Traditional Chinese Medicine Systems Pharmacology Database and Analysis Platform (TCMSP, https://old.tcmsp-e.com/tcmsp.php) and the compounds were supplemented according to the results of mass spectrometry and references (Zhang et al., 2016). To screen the chemical components identified above, one of the following conditions should be met: (1) Components whose oral availability (OB) ≥ 30% and drug-like (DL) ≥0.18; (2) Components which were quality markers specified in People’s Republic of China (PRC) Pharmacopoeia; (3) Components with related biological activity or high content reported in literature, or components that could enter the blood (Zheng et al., 2024). Subsequently, we retrieved the target of selected compounds in the HERB database (http://herb.ac.cn) and BATMAN-TCM database (http://bionet.ncpsb.org.cn). Then a herb-compound-target network was constructed with Cytoscape 3.10.2 software, based on the screened compounds and related targets.

#### 2.3.2 HN-associated microarray data analysis

Searching “hypertensive nephropathy” in the Gene Expression Omnibus (GEO) database (https://www.ncbi.nlm.nih.gov/geo/), the inclusion criteria were as follows: (1) human mRNA expression data set; (2) all samples consisted of tubulointerstitial tissue; (3) a case-control design was employed with sample sizes exceeding 3 for each group. After filtration, two data sets, GSE37460 and GSE99340 were yielded, which included 40 healthy individuals and 25 HN patients. The R package “SVA” and “limma” were used to adjust the batch effects between different datasets and the differentially expressed genes (DEGs) were screened with the adjusted *P*-value <0.05 as the screening condition. The DEGs were then subjected to gene ontology (GO), Kyoto Encyclopedia of Genes and Genomes (KEGG) by gene set enrichment analysis (GSEA) using the R package “clusterProfiler”.

To further study the role of mitochondria in HN, the mitochondrial protein database, MitoCarta3.0 (http://www.broadinstitute.org/mitocarta) (Rath et al., 2021), was visited to obtain 1,136 human mitochondria-localized genes and 149 mitochondria-associated gene enrichment pathway. The GSEA was carried out with the aid of the Mitocarta database to conduct a preliminary study of the biological function of the DEGs.

#### 2.3.3 Construction of PPI network and GO and KEGG enrichment analysis for treating HN with JYTL

Through importing the drug target genes and DEGs into the venny2.1 platform (https://bioinfogp.cnb.csic.es/), intersection genes were obtained. Then, the intersection genes were fed into STRING (https://string-db.org/) to construct the PPI network. The species “*Homo sapiens*” and the lowest interaction threshold “medium confidence (0.400)” were set. The data were visualized and the PPI network was plotted using Cytoscape software. In addition, the GO and KEGG pathway enrichment analysis was conducted using the Metascape database (https://metascape.org/), and the results were visualized.

### 2.4 Reagents

TGF-β1 antibody (ab92486), α-SMA antibody (ab5694), COLⅢ antibody (ab7778), OPA1 antibody (ab157457), Drp1 antibody (ab184247), SIRT1 antibody (ab189494), PGC-1α antibody (ab313559), Nrf1 antibody (ab175932), TFAM (ab307302), PINK1 antibody (ab186303), Parkin antibody (ab77924) and Beclin-1 antibody (ab207612) were purchased from Abcam (Cambridge, United Kingdom). Mfn1 antibody (sc-166644) and Mfn2 antibody (sc-100560) were purchased from Santa Cruz Biotechnology (Dallas, TX, United States). Mff antibody (17090-1-AP), Fis antibody (10956-1-AP), and P62 (18420-1-AP) were purchased from ProteinTech (Wuhan, China). GAPDH (G0100-1), HRP labeled Goat Anti-Rabbit IgG (S0101-1) and HRP labeled Goat Anti-Mouse IgG (S0100-1) were purchased from LABLEAD (Beijing, China).

### 2.5 Grouping and treatment

The SHRs were randomly divided into four groups with ten rats in each group: model group, JYTL group, Ex527 + JYTL group, and Valsartan group. The WKY rats were used for the control group. In our previous study, we have conducted dose-response studies of low, medium, and high doses of JiangyaTongluo decoction in SHRs and the dosages (14.2 g/kg/d JYTL by gavage) were determined from the pharmacodynamic experimental study (Han et al., 2015b). Valsartan [30 mg/kg/d, intragastrically (ig)] was administered in the valsartan group; The Ex527 + JYTL group was given Ex527 (2.5 mg/kg) by intraperitoneal injection half an hour after administration every other day. The rats were weighed every week, and the drug dosage was adjusted based on the weighing results. Triple-distilled water was applied to the rats in the WKY rats group and the model group and the intragastric administration volume was calculated to be 10 mL/kg/d.

After 12 weeks of treatment, metabolic cages were used for 24-h urine collection. Subsequently, the rats were sacrificed to collect the blood and kidney samples. The mass of both kidneys was weighed after sacrifice, and the kidney hypertrophy index was calculated by the ratio of kidney weight to body weight (KW/BW).

### 2.6 Blood pressure detection

Systolic blood pressure (SBP) and diastolic blood pressure (DBP) of the rat tail vein were measured using a noninvasive blood pressure meter (Softron Biotechnology Co., Ltd., Beijing, China, BP-98A) before treatment (0 week) and after 4, 8 and 12 weeks. Rats were trained for a week on the device before initiation of the experiment. All rats that participated in this experiment were fixed onto the operating table, and the pressure tail sleeve was placed in the appropriate position of the rat tail. After the rats were stabilized, the whole body was heated, the workstation was operated, and the SBP and DBP were measured and recorded. The information was continuously measured three times, and the average value was taken as a measurement value.

### 2.7 Measurement of renal function

The level of serum creatinine (Scr), blood urea nitrogen (BUN), blood uric acid (UA), and 24-h urinary total protein (24-h UTP)was examined by the Department of Laboratory Medicine of Beijing University of Chinese Medicine, using an automatic biochemistry analyzer (Beckman Coulter, AU5800).

### 2.8 Histological analysis

Kidney tissues of each animal were fixed with 10% formalin for more than 48 h, dehydrated, embedded in paraffin, and sectioned to a thickness of 5 µm. Then the slices were stained with hematoxylin-eosin (HE) and Masson staining. HE staining was used to analyze the general morphological changes of kidneys and Masson staining was used to assess the levels of collagen deposition and interstitial lesions. Representative structures of tissues were selected and photographed. A semi-quantitative analysis for collagen areas was performed using ImageJ.

### 2.9 Immunohistochemistry (IHC) staining

Deparaffinized sections (in xylene) were rehydrated in 100%, 95%, 90%, 80%, and 70% ethanol, respectively. Antigens were extracted in citrate buffer by boiling them for 30 min. Subsequently, the endogenous peroxidase activity was inactivated with 3% hydrogen peroxidase. Normal goat serum (5%) was used to prevent non-specific staining, and the primary antibodies were incubated overnight at 4°C, followed by horseradish peroxidase (HRP)-conjugated secondary antibodies (PV9001/PV9002, Beijing Zhongshan Jinqiao Biotechnology Co., Ltd., Beijing). Then, the sections were washed in PBS and stained in DAB and hematoxylin, respectively. Finally, the sections were rinsed with water, dehydrated in ethanol, clarified in xylene, and coverslipped (Li et al., 2023). Images were obtained by the optical microscope (Leica, Wetzlar, Germany). Image analysis and quantification were performed using ImageJ.

### 2.10 Detection of ROS

2′,7′-Dichlorodihydrofluorescein diacetate (DCFH-DA) fluorescence dye was used to detect the level of ROS in rat renal tissues according to the instructions provided by Shanghai Biyuntian Biotechnology Co., Ltd. (S0033S, China). In brief, 5-μm fresh frozen sections from the renal were incubated in DCFH-DA at 37°C for 30 min and observed by fluorescence microscopy (Nikon, ELCIPSE-CI, Japan). To quantify ROS generation, fluorescence intensity was measured using ImageJ.

### 2.11 Transmission electron microscopy assay (TEM)

Tissues from the kidney cortex of each rat were cut into 0.1 × 0.1 × 0.1 cm^3^ blocks and fixed in 2.5% glutaraldehyde (pH 7.4, Spi-Chem, United States) for 2 h. After being washed three times with 0.1 M phosphate buffer (pH 7.2) and fixed in 1% osmic acid (Ted Pella Inc., United States) at 4°C for 2 h, all the samples were gradient dehydrated in a graded series of ethanol. Subsequently, the samples were embedded in Epon-Araldite resin (Spi-Chem) for penetration and placed in a model for polymerization. After positioning, the ultrathin sections were collected for microstructure analysis. Counterstained using 3% uranyl acetate and 2.7% lead citrate (Wu et al., 2022). Finally, the samples were observed with an HT7800 transmission electron microscopy (TEM).

### 2.12 Adenosine triphosphate (ATP) content determination

The kidney tissue was homogenized (10% w/v) in ice-cold Phosphate buffer (50 mM, pH 7.4) and centrifuged at 3,000 rpm for 15 min at 4°C. The protein samples were quantified using a biochemical kit (Nanjing Jiancheng Bioengineering Institute, China). Then, according to the manufacturer’s instructions, the contents of ATP in renal tissue were measured using commercial kits (Nanjing Jiancheng Bioengineering Institute, China).

### 2.13 Western blot analysis

For Western blot assays, renal tissues were lysed and homogenized in RIPA buffer supplemented with protease inhibitor cocktail l (P1045, Beyotime, China) and quantified with a BCA kit (B5001, LABLEAD, China). Protein sample extracts (30 mg/lane) were separated by SDS-PAGE and transferred onto a polyvinylidene difluoride membrane (PVDF). After the membranes were blocked with 5% BSA, they were incubated with the primary antibodies at 4°C overnight, followed by HRP-conjugated secondary antibody. Then, the membranes were incubated with horseradish peroxidase-conjugated goat anti-mouse or anti-rabbit IgG (1:5,000, LABLEAD, China) for 1 h at room temperature. Films were scanned by a ChemiScope 6,000 system (Qinxiang, Shanghai, China). ImageJ software was used to measure the protein bands based on that of GAPDH.

### 2.14 Statistical analysis

Data are expressed as means ± SEM. The significance of the differences in mean values between multiple groups was examined by analysis of variance (one-way *ANOVA*). To compare between every two groups, the *LSD* t-test or Tamhane’s test was used. All statistical analyses were performed using SPSS26.0 statistical software. *P*  < 0.05 was considered statistically significant.

## 3 Results

### 3.1 Identification of absorbed components of JYTL in rat serum based on UPLC-QE/MS

**FIGURE 1:**
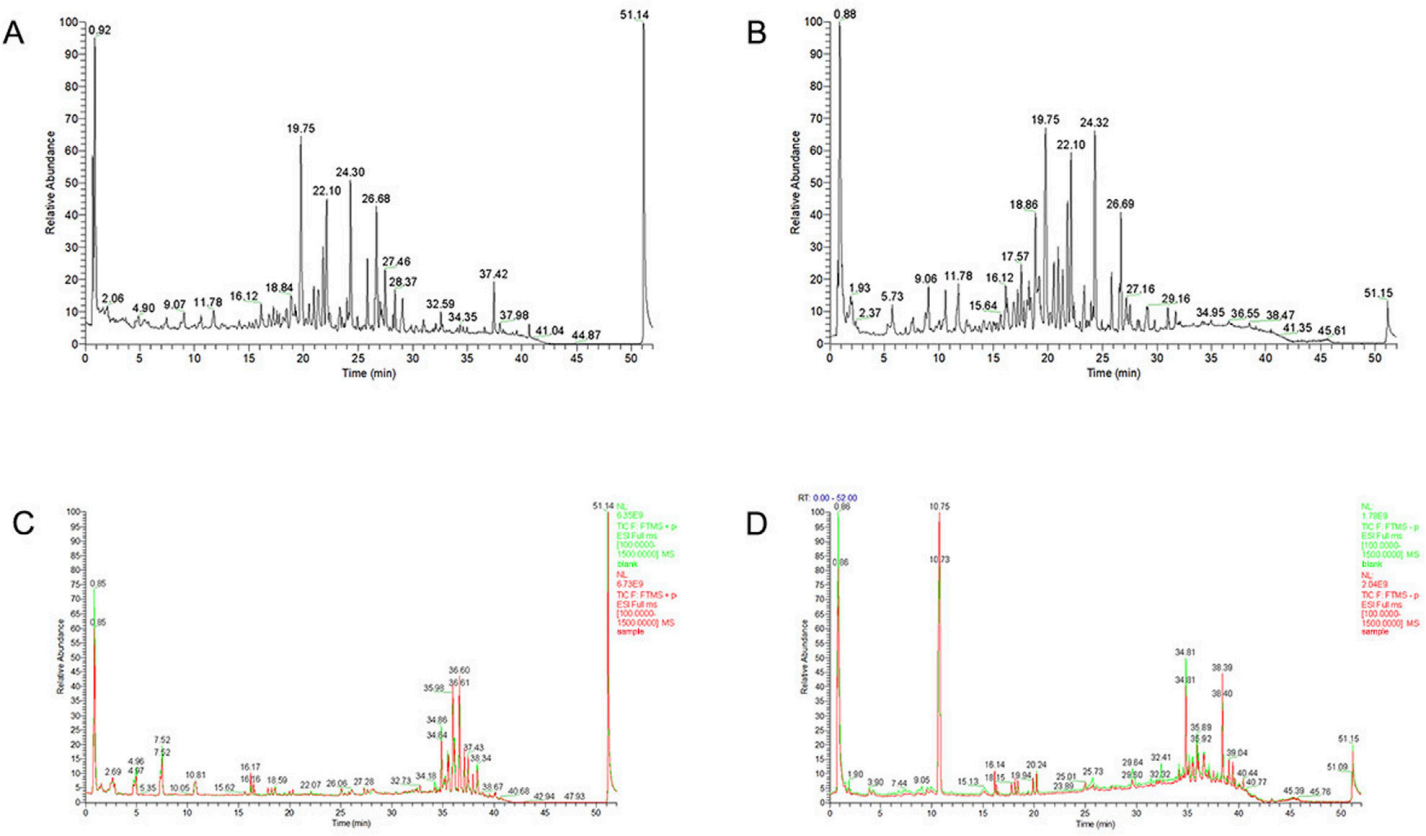
Chemical ingredients analysis of JYTL decoction. Total ion chromatography in positive **(A)** and negative **(B)** ion modes for JYTL samples and blank serum and dosed serum samples in positive **(C)** and negative **(D)** modes are shown. Among them, red represents administered serum, and green represents blank serum.

The major chemical components in JYTL freeze-dried powder were analyzed by UPLC-QE/MS in positive and negative ion mode (Figures 1A, B). A total of 178 constituents in JYTL were tentatively identified (Supplementary Table S1). Further, chemical profiling of serum obtained after JYTL administration was performed to characterize the absorbed components to find possible therapeutic compounds of JYTL decoction. After 12 weeks of treatment as described in method Section 2.5, serum of JYTL group and model group were collected. Compared with the model group, 31 components were detected in SHRs’ serum following JYTL treatment, mainly including epicatechin, trans caffeic acid, azelaic acid, baicalin, formononetin, baicalein, and other natural compounds. The detailed information is shown in Figures 1C, D, and Supplementary Table S2.

### 3.2 Network pharmacology analysis

**FIGURE 2:**
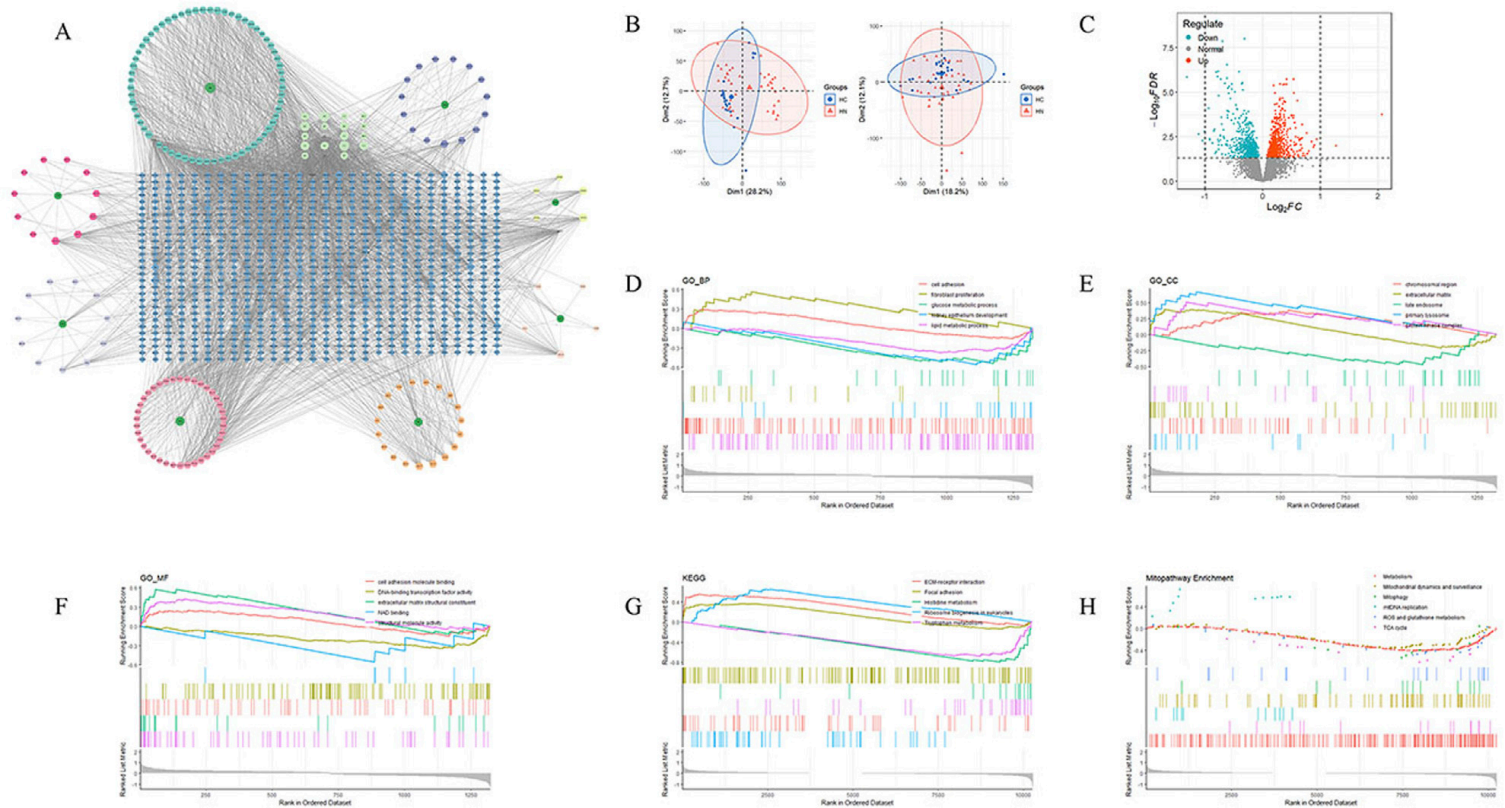
Target genes of active ingredients in JYTL and HN-associated microarray data analysis. **(A)** Network analysis depicting the relationship between JYTL components and their target genes. **(B)** Batch effect before and after correction. **(C)** Volcano plot of differentially expressed genes, with red indicating upregulation of differential expression and blue indicating downregulation of differential expression. **(D–F)** Results of GSEA of gene ontology (GO) for biological processes (BP), cellular components (CC), and molecular functions (MF) of differentially expressed genes. **(G)** KEGG pathway enrichment analysis of differentially expressed genes. **(H)** Annotation of significant MitoPathways by GSEA analyses.

Through network pharmacology analysis, a total of 189 active ingredients of JYTL were screened, and target prediction based on the HERB and BATMAN-TCM database resulted in 804 predicted targets. The active ingredients and related targets were imported into Cytoscape 3.10.0 software to construct the herb-active ingredient-target network (Figure 2A). Using the GEO database, a total of 1,324 DEGs were identified in HN tubulointerstitial tissue, including 715 upregulated and 609 downregulated genes (Figure 2C). The microarrays were corrected employing the SVA algorithm, and PCA plots were used to demonstrate the batches before and after correction (Figure 2B). The results of GSEA on all DEGs demonstrated that in the renal tissue gene expression matrix of HN patients, active GO functions are mainly enriched in chromosomal region, extracellular matrixlate, late endosome, primary lysosome, protein kinase complex, cell adhesion, fibroblast proliferation, glucose metabolic process, kidney epithelium development, lipid metabolic process, cell adhesion molecule binding, DNA-binding transcription factor activity, extracellular matrix structural constituent, NAD binding, structural molecule activit, etc. (Figures 2D–F). The most enriched KEGG pathways of the DEGs were dominated by pathways involved in ECM-receptor interaction, focal adhesion, histidine metabolism, ribosome biogenesis in eukaryotes, and tryptophan metabolism (Figure 2G). Subsequently, to explore the association between HN and mitochondria, we performed GSEA of differentially expressed genes (Figure 2H). As a result, the DEGs may be correlated with mitochondrial dynamics and surveillance, mitophagy, mtDNA replication, oxidative stress, and tricarboxylic acid cycle (TCA cycle).

**FIGURE 3:**
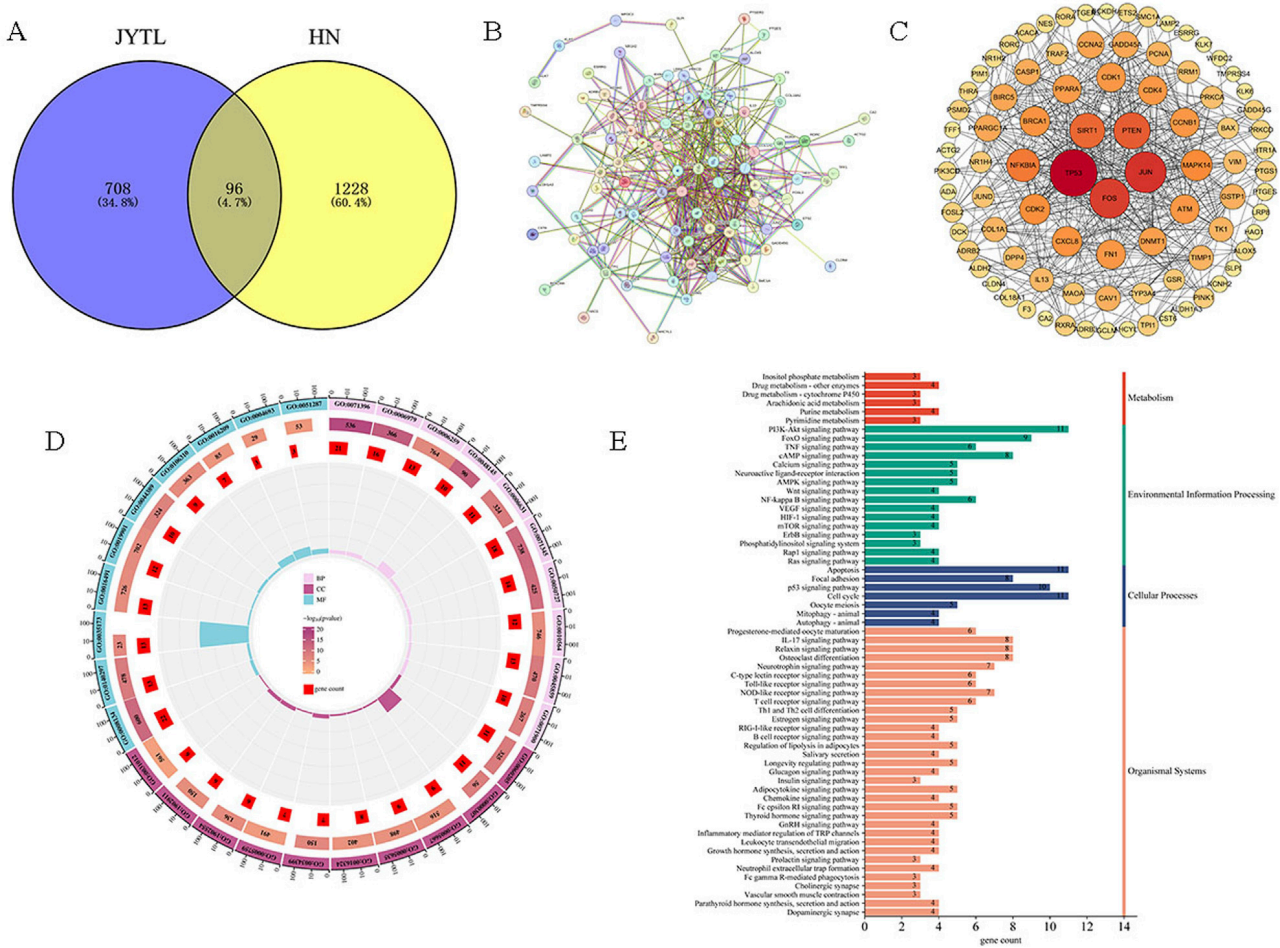
Prediction of disease targets of JYTL in HN through network pharmacology. **(A)** The common target genes of JYTL and HN. **(B, C)** PPI network for common targets. The darker the color, the greater the degree value, indicating the gene was more likely to be the core gene. **(D)** Enrichment analysis of GO for BP, CC, and MF of common genes. **(E)** KEGG pathway enrichment analysis of common genes.

Next, to further study the potential action mechanisms of JYTL against HN, 96 genes were obtained by intersecting differentially expressed genes with the targets of JYTL active ingredients (Figure 3A). A PPI network consisting of 96 nodes and 499 edges was obtained using the STRING database (Figure 3B). It was then visualized using Cytoscape (Figure 3C). The PPI network analysis revealed that the central cross-targets of TP53, JUN, FOS, PTEN and SIRT1 might be the most important potential targets of JYTL for the treatment of HN (degree >30). Meanwhile, the 96 intersecting targets were analyzed for GO function and KEGG pathway enrichment using the Metascape database. The results of the GO enrichment analysis (Figure 3D) indicated that biological processes (BP) were primarily enriched in cellular response to lipid, regulation of protein kinase activity, response to oxidative stress, regulation of inflammatory response, cellular response to cytokine stimulus, regulation of fibroblast proliferation, DNA metabolic process, regulation of cell cycle process, fatty acid metabolic process and regulation of protein serine/threonine kinase activity. Key cellular components (CC) included mitochondrial matrix, serine/threonine protein kinase complex, and protein kinase complex. In terms of molecular functions (MF), the targets were mainly associated with DNA-binding transcription factor binding, oxidoreductase activity, protein kinase binding, protein serine kinase activity, antioxidant activity, cyclin-dependent protein serine/threonine kinase activity, and NAD binding. KEGG pathway analysis (Figure 3E) revealed associations with pathways such as “Apoptosis”, “PI3K-AKT signaling pathway”, “IL-17 signaling pathway” and “Mitophagy”.

### 3.3 Effect of Jiangya Tongluo decoction on blood pressure and renal function in SHRs

**FIGURE 4:**
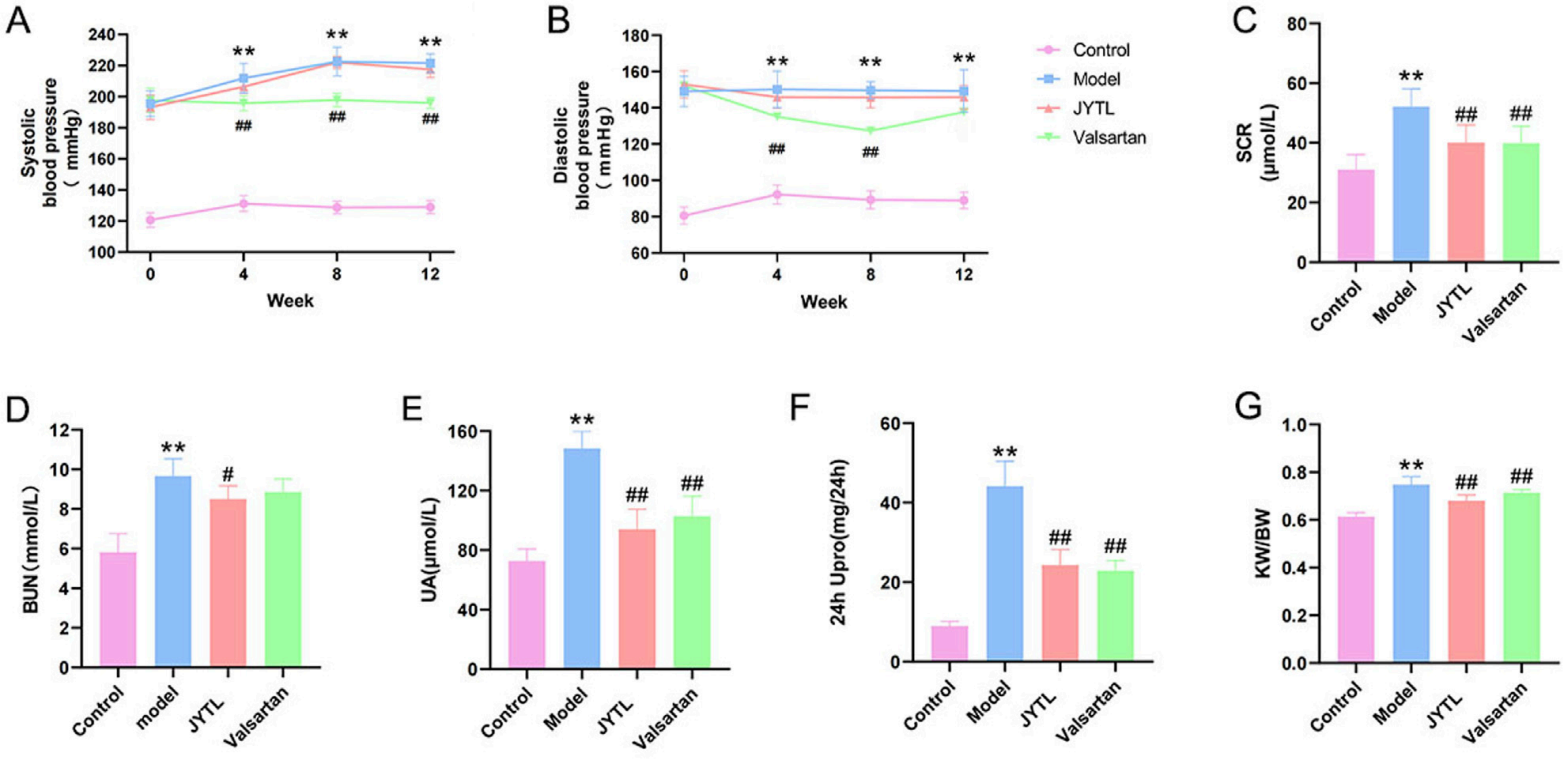
Effect of JYTL Decoction on blood pressure and renal function in SHRs. Changes of Systolic blood pressure **(A)** and Diastolic blood pressure **(B)** in different groups since the start of the gavage intervention; The detection of Scr **(C)**, BUN **(D)**, UA **(E)**, and 24-h urinary protein quantification **(F)** levels in circulation (n = 8); **(G)**. Comparisons of renal index among different groups (n = 8), renal index = kidney weight **(G)**/body weight **(G)**. Data were presented as means ± SEM. Compared to the WKY rats group, **p < 0.01, compared to the model group, #*p* < 0.05, ##*p* < 0.01. The * and ^#^ in the figure below represent the same comparisons.

To confirm the therapeutic effects of JYTL decoction on hypertensive nephropathy, we adopted SHRs within 12 weeks of JYTL treatment and tested blood pressure and kidney function. As shown in Figures 4A, B, systolic and diastolic blood pressure of rats in the model group and both treatment groups were significantly higher than in the WKY rats group (*P* < 0.01) prior to treatment, indicating that the model was successfully established. At 4 weeks, 8 weeks, and 12 weeks after drug administration, the systolic and diastolic blood pressure in the rats of the JYTL group decreased compared to the age-matched model group, however, the decrease showed no statistical difference (all *P* > 0.05).

Persistent hypertension has been shown to contribute to kidney damage. After 12 weeks of treatment, we found that the SHR group had higher levels of Scr (52.11 μmol/L), BUN (9.67 mmol/L), UA (148.40 μmol/L), and 24 h UPro (44.13 mg/24 h) than the WKY group (30.95 μmol/L, 5.81 mmol/L, 72.67 μmol/L, 8.95 mg/24 h respectively) (all *P* < 0.001), whereas these renal-function-related markers were significantly lower in the JYTL groups (Scr: 40.03 μmol/L, BUN: 8.5 mmol/L, UA: 93.95 μmol/L and 24 h UPro: 24.28 mg/24 h) than in the SHR group (all *P* < 0.001) (Figures 4C–F). Similarly, the level of SHRs KW/BW (0.75) was significantly higher than that in the WKY group (0.62), and JYTL treatment partly decreased the levels to 0.68 (Figure 4G). As a positive control group, Valsartan decreased the level of Scr, UA, 24 h UPro, and KW/BW (*p* < 0.05) but not BUN.

### 3.4 Jiangya Tongluo decoction attenuated pathologic changes of renal tissues in the rats with hypertensive nephropathy

**FIGURE 5:**
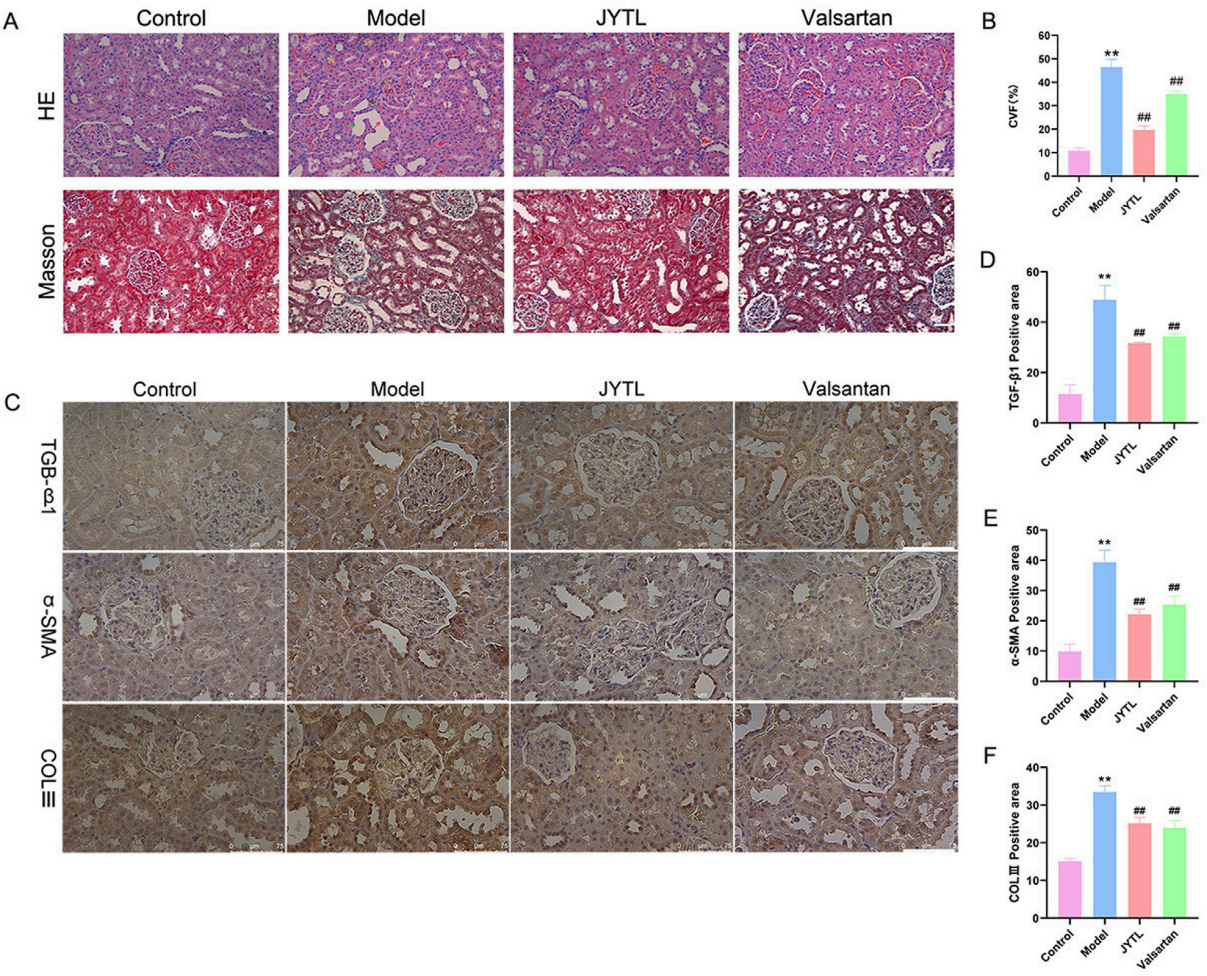
JYTL decoction treatment attenuated renal injury in SHRs. **(A)** H&E and Masson’s trichrome were performed to evaluate the general morphological changes of kidneys. The magnification of the images is ×400, scale bar = 50 μm. **(B)** A semi-quantitative analysis of the collagen areas according to Masson’s trichrome staining. **(C–F)** Expression levels of TGF-β1, a-SMA, and COLⅢ in the kidney were detected by immunohistochemistry, and the optical intensity of the abovementioned proteins was measured (n = 6). The magnification of the images is ×400, scale bar = 75 μm. Data were presented as means ± SEM (n = 6).

Histological changes in the kidneys of rats treated with different therapeutic regimens were examined using HE and Masson’s trichrome staining (Figures 5A, B). Results from HE staining showed that there were glomerular ischemia and sclerosis (red five-pointed stars); interstitial fibrosis with inflammatory cell infiltration (yellow rhombus); compensatory dilatation (green arrow), loss of brush border and vacuolar degeneration (yellow five-pointed stars) in renal tubules; flattening and exfoliation of epithelial cells (red arrows mark). However, JYTL and Valsartan administration mitigated these pathological lesions. As Masson’s trichrome analysis showed, SHR promoted renal fibrosis compared with the normotensive WKY rats, which was significantly reduced in JYTL and Valsartan groups (all *p* < 0.01), suggesting that JYTL alleviated renal fibrosis.

To further investigate the protective effects of JYTL on renal fibrosis, we investigated the expression of TGF-β1, α-SMA, and COLⅢ by immunohistochemistry. As shown in Figures 5C–F, compared with the WKY rats group, the SHR group showed a dramatic increase in TGF-β1, α-SMA, and COLⅢ levels in the tubular interstitium, while JYTL treatment significantly inhibited this abnormal increase. Similarly, valsartan treatment significantly reduced the level of TGF-β1, α-SMA, and COLⅢ.

### 3.5 Jiangya Tongluo decoction inhibits mitochondrial dysfunction in SHRs by upregulating SIRT1

**FIGURE 6:**
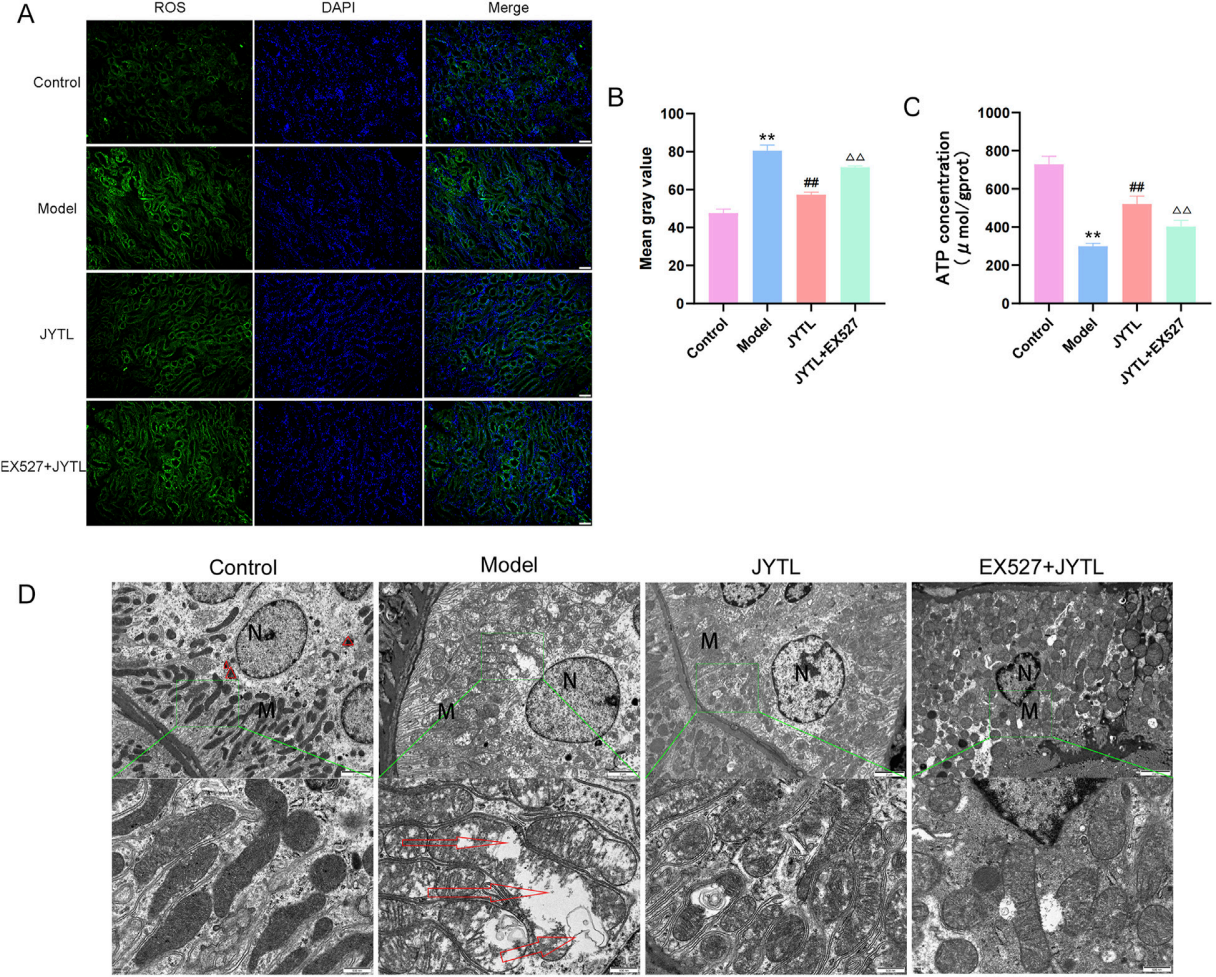
JYTL improves mitochondrial function and morphology. **(A)** DCFH-DA fluorescent staining for detecting reactive oxygen species (ROS). **(B)** Image-J-based quantification of DCFH-DA fluorescent intensity. Data are shown as the means ± SD from three independent experiments.^△△^ denote statistical significance at the *P* < 0.01 level compared to the JYTL group. **(C)** ATP content in rat renal tissues. The data is represented by Mean ± SD (n = 6). **P < 0.01 versus the WKY rats group; ^##^ *P* < 0.01 versus the model group.^△^ *P* < 0.05, ^△△^ *P* < 0.01 versus the JYTL group. **(D)** Transmission electron microscopy (TEM) images of mitochondrial morphology in the renal cortex following pharmacological intervention (n = 3). The red triangles indicate mitophagosomes. Green-boxed areas are enlarged and presented in the bottom panel. Red arrows indicate damaged mitochondria. Scale bars: 2 μm (top panel); 500 nm (bottom panel).

DCFH-DA, a green fluorescent probe, was conducted to evaluate ROS production in the kidneys. As illustrated in Figures 6A, B, ROS production was significantly greater in the kidneys of SHRs than in those of WKY rats. Furthermore, the level of ROS decreased significantly following JYTL treatment. Subsequently, we probed into whether SIRT1 is involved in JYTL-mediated renal protection in SHRs. Thirty minutes after JYTL gavage therapy, SHRs received Ex527, a pharmacological inhibitor of SIRT1. As a result, ROS production in the Ex527 + JYTL group was aggravated compared to the JYTL group (Figures 6A, B), suggesting that the blockade of SIRT1 offsets the protective effect of JYTL against hypertensive kidney lesions. ATP content changes reflect the degree of mitochondrial damage inside the cell. As shown in Figure 6C, the results revealed that, compared with those in the WKY group, ATP levels in the SHR group were markedly reduced, and ATP levels were increased after JYTL treatment, while SIRT1 inhibition reversed the protective effect of JYTL against mitochondrial dysfunction in the kidney.

In addition, we used transmission electron microscopy to observe the structure of the mitochondria in renal tubular epithelial cells (RTECs). TEM showed many swollen mitochondria in RTECs with broken, dissolved, or even disappeared cristae in the SHR group, while the JYTL treatments relieved the degree of mitochondrial swelling and increased the number of autophagosomes. In the Ex527 + JYTL group, the slight swelling of the cells remained, and the mitochondrial swelling was relieved but still common, upon comparison with the JYTL group, the expansion, fracture and disintegration of the cristae were more serious to some extent (Figure 6D).

**FIGURE 7:**
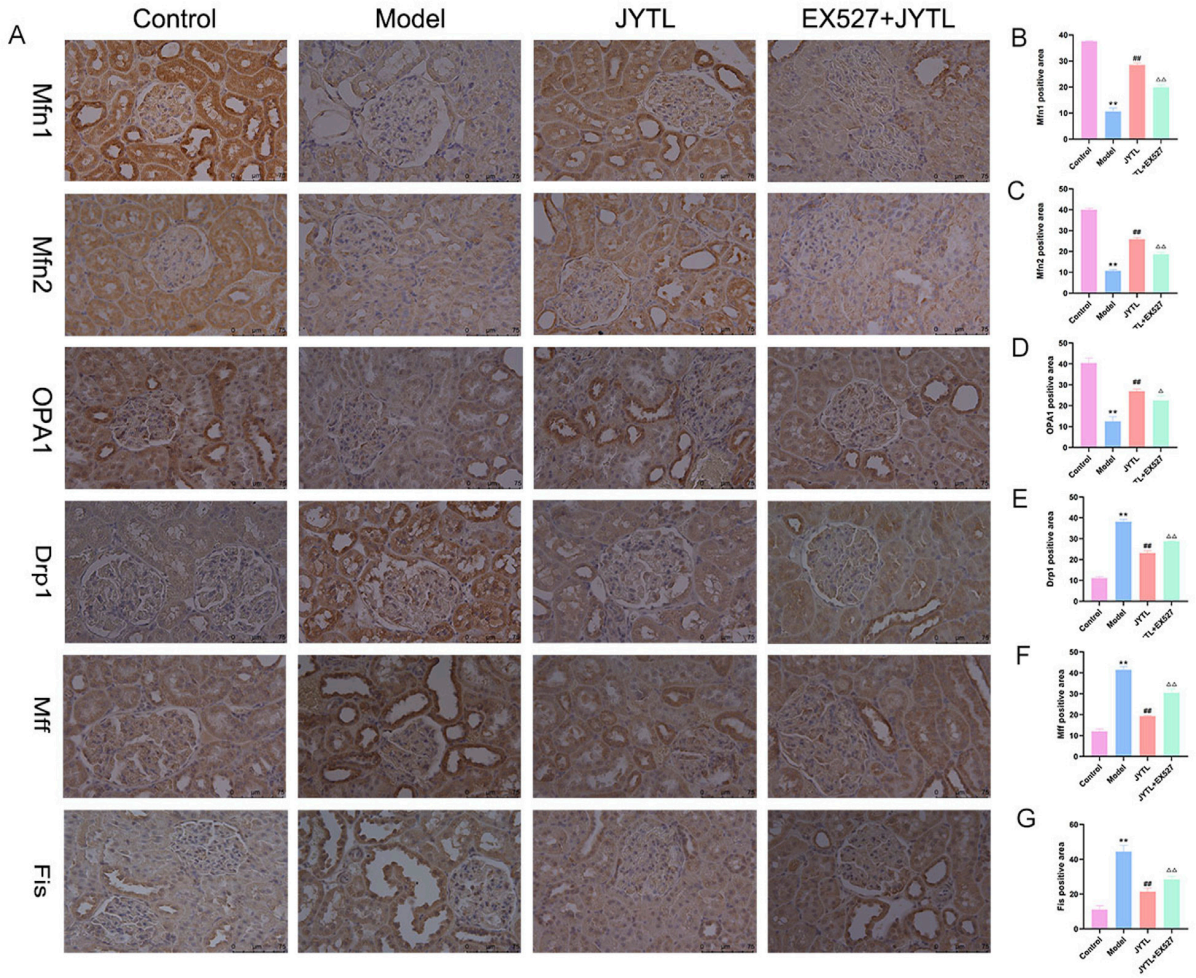
JYTL decoction modulated the expression of proteins involved in mitochondrial dynamics. **(A)** Expression levels of mitochondrial fusion protein Mfn1, Mfn2, and OPA1 and mitochondrial fission protein Drp1, Mff, and Fis in the kidney were detected by immunohistochemistry. **(B–G)** The analytical result of the abovementioned proteins was measured. The magnification of the images is ×400, scale bar = 75 μm. Data were presented as means ± SEM (n = 6). **P < 0.01 versus the WKY rats group; ^##^ *P* < 0.01 versus the model group.^△^ *P* < 0.05, ^△△^ *P* < 0.01 versus the JYTL group.

Mitochondrial division and fusion play critical roles in maintaining morphology and function. Previous studies have highlighted the presence of excessive mitochondrial fission and fragmentation during the progression of HN (Ko et al., 2023). Therefore, we investigated the expression of proteins associated with mitochondrial dynamics (Figure 7A). Consistent with our expectations, levels of the mitochondrial fusion proteins Mfn1, Mfn2, and OPA1 significantly decreased in the renal tissue of the model group (Figures 7B–D). However, JYTL treatment reversed this decrease in all fusion protein levels. Conversely, levels of the mitochondrial fission-related proteins DRP1, Mff, and Fis significantly increased in SHR kidney tissue. Once again, JYTL intervention had a corrective effect, effectively reducing the levels of these fission proteins to normal levels (Figures 7E–G). Additionally, in the Ex527 + JYTL group, mitochondrial fusion proteins significantly declined and the fission proteins were increased compared with the JYTL group. These findings suggested that JYTL intervention protected against mitochondrial dysfunction in SHRs with hypertensive nephropathy.

### 3.6 Jiangya Tongluo decoction promotes mitochondrial biogenesis in SHRs with hypertensive nephropathy

**FIGURE 8:**
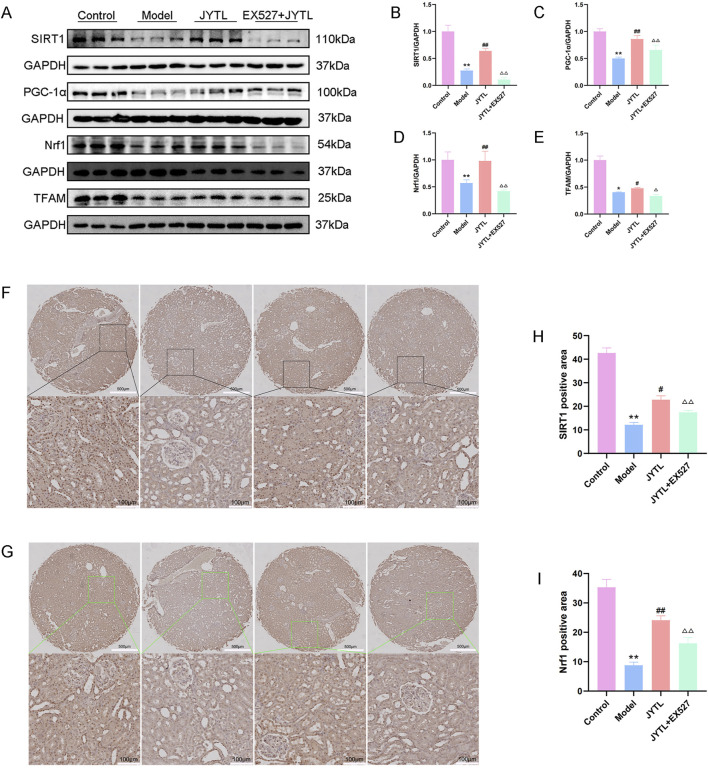
JYTL decoction promotes mitochondrial biogenesis in SHRs. **(A–E)** The expression of SIRT1, PGC-1α, Nrf1, and TFAM by Western blot (n = 3) and analyzed semi-quantitatively. Data are presented as mean ± standard deviation and analyzed using *one-way ANOVA*, followed by Tukey’s multiple comparison test for the *post hoc* test. **(F–I)** Levels of SIRT1 **(F)** and Nrf1 **(G)** in the renal cortex of rats were detected using immunohistochemistry. The boxed areas are enlarged and presented in the bottom panel. The magnification of the images is ×5 (top panel) and ×200 (bottom panel). Scale bars: 500 μm and 100 μm respectively. Data are expressed as means ± SEM; **P < 0.01 versus the WKY rats group; ^#^ *P* < 0.05, ^##^ *P* < 0.01 versus the model group.^△△^ *P* < 0.01 versus the JYTL group.

The role of JYTL in mitochondrial biogenesis of hypertensive renal damage progression was verified by Western blotting assay and immunohistochemical staining. The results revealed that compared with the WKY rats group, the relative expression of mitochondrial biogenesis-related proteins (SIRT1, PGC-1α, NRF1, and TFAM) decreased in the model group and the expression of these proteins reversed with the treatment of JYTL. However, Ex527 pretreatment abolished the protective effect of JYTL in the kidney of SHRs (Figures 8A–E). Immunohistochemical results were consistent with the Western blotting analysis (Figures 8F–I).

### 3.7 Jiangya Tongluo decoction promotes PINK1/Parkin-mediated mitophagy by activating the SIRT1 signaling

**FIGURE 9:**
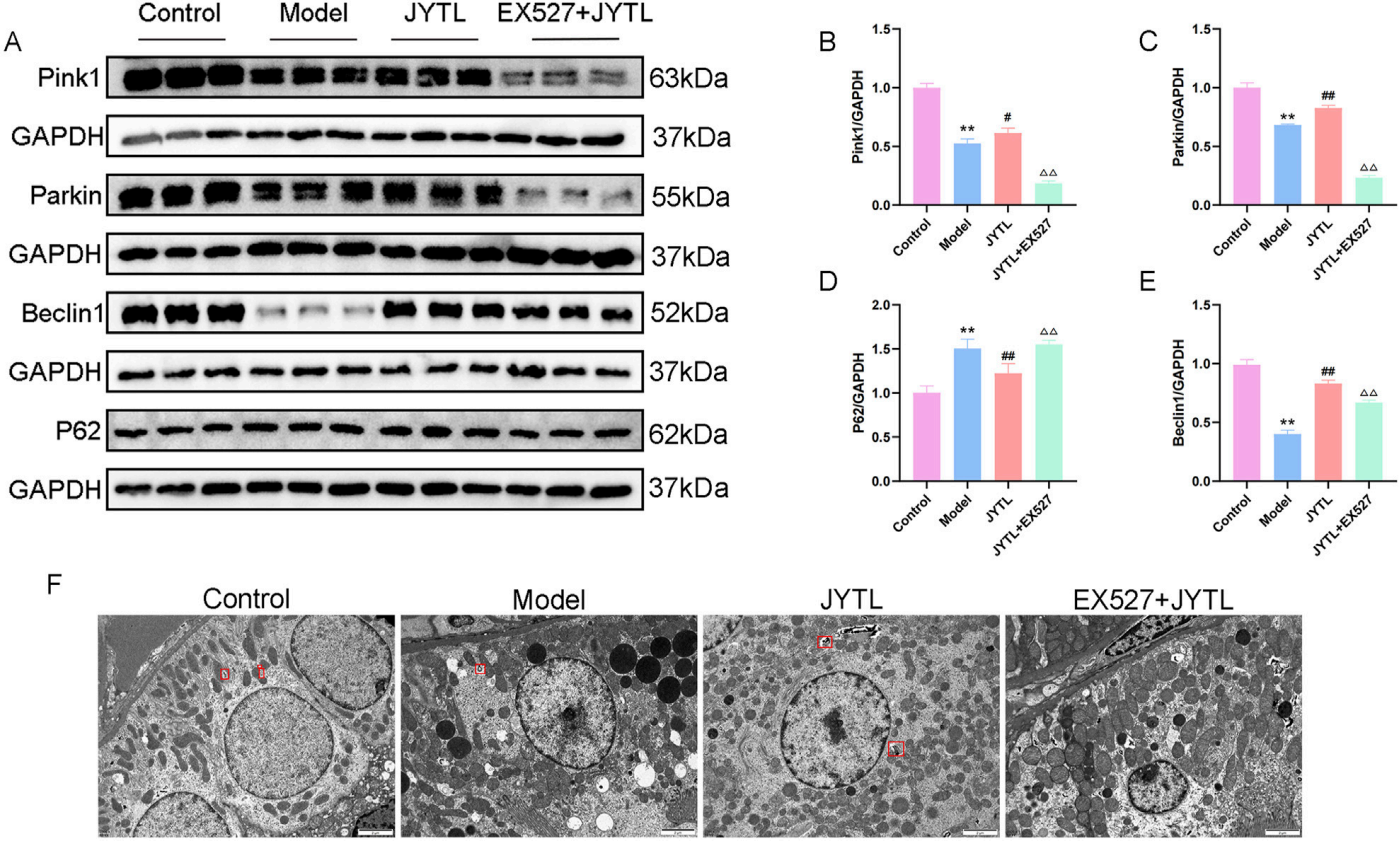
JYTL decoction promotes PINK1/Parkin-Mediated Mitophagy by Activating the SIRT1 signaling. **(A–E)** Representative Western blot analysis of PINK1, Parkin, Beclin1, and P62 in kidney tissue (n = 3). **(F)** Representative TEM images of mitophagy in rats (n = 3). Red quadrilateral indicated autophagosomes (scale bar: 2 μm).

To address further whether Jiangya Tongluo decoction-induced activation of SIRT1 is required for mitophagy induction and the underlying mechanisms, we evaluated mitophagy of kidney tissue in the presence or absence of Ex527. The expression of proteins related to mitophagy signaling, including PINK1, Parkin, Beclin1, and p62 were analyzed by Western blotting. Our results showed significantly higher levels of PINK1, Parkin, and Beclin1 and lower levels of p62 in the JYTL group as compared with the model group, while Ex527 attenuated this effect (Figures 9A–E).

Furthermore, we used transmission electron microscopy to observe the quantity of the mitochondria and autophagosomes. As shown in Figure 9F, there are abundant mitochondria with more autophagosomes (red quadrilateral) in the WKY rats group. However, the integrity of the mitochondria was disrupted and was accompanied by fewer autophagosomes in the model group. Interestingly, Jiangya Tongluo decoction increased the quantity of the mitochondria and autophagosomes. Moreover, we found no mitophagosome in all fields of view in the Ex527 + JYTL group, indicating addition of the SIRT1 inhibitor significantly reduced mitophagy, and damaged mitochondria accumulated but not eliminated. Together with the results, it is suggested that the mechanism by which Jiangya Tongluo decoction treatment increases mitophagy involves the SIRT1-PINK1-Parkin pathway.

## 4 Discussion

In recent years, hypertension has been the second or third most common cause of renal replacement therapy (RRT) in Europe, tied with glomerulonephritis (Boerstra et al., 2024). Hypertensive nephrosclerosis is also the second most frequent cause of RRT in the United States and the third in China (Johansen et al., 2024; Yang et al., 2020). The renal injury caused by hypertension is characterized by renal tubular injury, renal interstitial fibrosis, and glomerulosclerosis (Yao et al., 2023). Compelling evidence demonstrated that the proximal tubule plays a central role in renal fibrosis (Gewin, 2018). In the course of TIF, the activation of fibroblasts and overproduction and deposition of extracellular matrix (ECM) contribute to the destruction of renal parenchyma and the progressive loss of renal function (Kuppe et al., 2021). The SHR provides an opportunity to study essential hypertension, as the natural progression of hypertension and organ damage including the kidneys is remarkably similar to humans (Cai et al., 2021; Chandran et al., 2014). In this study, the Scr, BUN, UA, 24 h-UTP and kidney index, which are indicators of impaired kidney significantly increased in the SHR group compared to that in the WKY rats group. Meanwhile, TIF characterized by significantly elevated expression levels of TGF-β1, α-SMA, and COLⅢ was detected in the model group. These results were consistent with previous research, indicating that the animal model is consistent with the typical features of hypertensive renal damage (Tang et al., 2011; Song et al., 2024; Huang et al., 2018).

Due to the multi-target function, TCM treatment shows promising clinical benefits as the main or alternative therapy for HN treatment (Huan et al., 2023; Lu Y. et al., 2024). The academic thoughts and clinical experience of famous veteran TCM doctors were important content of TCM (Ren et al., 2023). JYTL was created according to Guo’s medical school on the treatment of hypertension and the application of the method of promoting blood circulation and removing blood stasis (Qin et al., 2015; Ma et al., 2016). JYTL decoction is composed of eight herbs, and 31 absorbed compounds were identified by UPLC-QE/MS analysis. Studies have proved that azelaic acid, Baicalin, and Trans caffeic acid can effectively ameliorate TIF (Muthulakshmi and Saravanan, 2013; Wang et al., 2022; Hsieh et al., 2022). It was also reported that epicatechin, through scavenging the increased levels of ROS in arsenic-exposed cells, has protective effects against fibrogenic changes in kidney epithelial cells (Iheanacho et al., 2024). Furthermore, 5-hydroxymethylfurfural can relieve the sickle cell trait, which is associated with increased risk for the common conditions of chronic kidney disease and venous thromboembolism (Hansen et al., 2020). In addition, tanshinone IIA could be considered a microvascular-protective drug that alleviates acute cardiac microcirculation IR injury by blocking mitochondrial damage (Zhong et al., 2019). Cryptotanshinone treatment improved mitochondrial function by inducing mitochondrial biogenesis, and increasing mitochondrial mass and DNA content (Imran et al., 2017). These protective effects and mechanisms are consistent with our observations in this study. These ingredients may be involved in the protective effect of JYTL in TIF.

In recent years, research on TCM formulas has evolved to reveal active ingredients and their mechanisms of action (Chen X. et al., 2024). Through network pharmacology, a comprehensive component-target network of JYTL was constructed, consisting of 189 components and 804 targets. The findings revealed that most compounds in JYTL were influenced by multiple targets. Notably, tanshinone iia, quercetin, and adenosine were identified as the main components, with 204, 154, and 113 target genes, respectively. Researches have shown that tanshinone iia attenuated cardiac microvascular ischemia-reperfusion injury via regulating the SIRT1-PGC1α-mitochondrial apoptosis pathway (Zhong et al., 2019). As a flavonoid with promising therapeutic applications, attention has been placed on the effect of quercetin on an array of mitochondrial processes (De Oliveira et al., 2016; Carrillo-Garmendia et al., 2024). Moreover, adenosine could enhance mitochondrial homeostasis, antioxidant defense, and autophagic flux to protect cardiomyocytes against acrolein-induced cardiotoxicity (Gao et al., 2024). These main components can regulate mitochondrial function. It is well known that the kidney is the organ with the second highest mitochondria abundance following the heart (Xiong et al., 2023). Mitochondrial dysfunction is considered a vital role in the pathogenesis of renal diseases (Carrillo-Garmendia et al., 2024; Bhargava and Schnellmann, 2017). Therefore, in the following study, we focused on the regulatory effect of JYTL on mitochondrial function in hypertensive nephropathy.

The GSEA analysis of the GEO and mitochondrial protein database shows that mitochondrial dynamics and surveillance, mitophagy, mtDNA replication, oxidative stress, and TCA cycle may be involved in the progression of HN. Similarly, an analysis of functional enrichment showed that the intersection targets of JYTL and HN are enriched in various pathways, including “Apoptosis”, “PI3K-AKT signaling pathway”, “IL-17 signaling pathway” and “Mitophagy”. Furthermore, the PPI network analysis of JYTL in treating HN identified TP53, JUN, FOS, PTEN, and SIRT1 as potential key genes involved in its therapeutic mechanism. Accumulating evidence has demonstrated that SIRT1 regulates oxidative stress and mitochondrial homeostasis in a variety of kidney diseases, including HN (Liu et al., 2022; Jin et al., 2023; Lu J. et al., 2024). Based on the results of GEO datasets, network pharmacology, and literature research, we focused on the regulation of mitochondrial biogenesis and mitophagy by SIRT1 to maintain mitochondrial homeostasis in HN and the intervention role of JYTL.

In this study, we found that JYTL plays a crucial role in improving renal function in SHRs, but the antihypertensive effect was not obvious. TIF is a dynamic and converging process, the core event of TIF is that the inflammatory microenvironment formed after renal injury activates myofibroblasts to produce a matrix (Peng et al., 2023). α-SMA is a marker protein of myofibroblasts, and TGF-β1 is a key mediator in myofibroblast formation, proliferation, and ECM production, such as collagen type Ⅲ (Col Ⅲ). In this study, α-SMA, TGF-β1, and Col Ⅲ levels were significantly lower in the JYTL group than in the model group, indicating that JYTL might alleviate TIF through its anti-fibrotic effect.

Current research findings compellingly advocate a substantial escalation in ROS production in the context of renal fibrosis (Zhao et al., 2023). The cumulative body of theories posits a direct link between the overproduction of ROS within mitochondria and ensuing mitochondrial dysfunction, ultimately resulting in RTECs damage and the progression of TIF (Zhao et al., 2024). In this study, we found that JYTL decoction effectively reduced the level of ROS in kidney tissues of SHRs. Meanwhile, the morphology of mitochondria in RTECs was observed by TEM, we found that the mitochondria in the model group were severely damaged, showing swollen and enlarged mitochondria, broken mitochondrial cristae, and formation of vacuoles, whereas JYTL treatment reversed this condition.

Moreover, mitochondria are exceedingly dynamic organelles that constantly undergo fusion/fission and turnover through mitochondrial biogenesis and mitophagy (Zhao et al., 2023). Mitochondrial fusion mitigates stress by joining partially damaged mitochondrial contents as a form of complementation, while fission is necessary to remove damaged mitochondria via the segregation of dysfunctional mitochondria (Meyer et al., 2017). However, excessive mitochondrial fission can lead to mitochondrial fragmentation and cause abnormal accumulation of mitochondrial ROS, which in turn impairs mitochondrial function. Our results indicated that there was a dynamic imbalance of mitochondria (upregulated Drp1, Mff, and Mff expression, downregulated OPA1, Mfn1, and Mfn2 expression) in the SHR group. Interestingly, JYTL treatment could preserve the stability of mitochondrial dynamics. Briefly, the above results confirmed our conjecture that JYTL decoction effectively alleviates the excessive division of mitochondria and promotes the fusion of mitochondria in SHRs, thus improving the dynamics of mitochondria, inhibiting excessive ROS production and maintaining the morphology and function of mitochondria. However, the abnormal mitochondria clearance and the regulatory effects of JYTL on mitophagy need further study.

SIRT1, the best-studied Sirtuin protein family member, is an NAD^+^-dependent deacetylase involved in multiple cellular functions related to mitochondrial energy homeostasis and antioxidant activity (Wakino et al., 2015) In this study, we focused on the key role of SIRT1 and investigated the related mechanism underlying the protective effect of JYTL in improving mitochondrial function. The advantageous effect of JYTL on mitochondria protection was reversed by the SIRT1 antagonist, Ex527, which further indicated that JYTL elicited kidney protection by activating the SIRT1 signaling pathway. Furthermore, SIRT1 could deacetylate PGC-1α, a transcriptional coactivator, which is important for mitochondrial biogenesis and mitochondrial function. PGC-1α can interact with NRF1 and promote its expression, thereby increasing downstream TFAM expression and promoting mtDNA replication. It was recently shown that SIRT1-PGC-1α-TFAM expression downregulation was observed in aldosterone-induced epithelial-to-mesenchymal transition of renal proximal tubular epithelial cells (Yuan et al., 2012). In this study, we have confirmed that JYTL promotes mitochondrial biogenesis by regulating Sirt1 to activate PGC-1α-NRF1-TFAM, which can help improve mitochondrial dysfunction. At the same time, our analysis revealed that EX527 reversed the aforementioned protective effects.

It is well known that mitochondria are highly dynamic and undergo constant turnover through two complementary processes: mitophagy, which selectively removes superfluous and damaged mitochondria, and reduces the source of oxidative stress, and mitochondrial biogenesis, which generates fresh, functional ones (Pickles et al., 2018). Proper coordination of mitophagy and mitochondrial biogenesis is essential for the quantity and quality control of mitochondria (Palikaras et al., 2015). Substantial evidence has reported that the activation of the SIRT1/PGC-1α pathway can increase the level of mitophagy (Liang et al., 2020; Han et al., 2023; Liu et al., 2021). Accelerating mitophagy during mitochondrial biogenesis may prevent over-burdening or over-crowding of the cell with excessive mitochondria, thus maintaining normal mitochondrial and organismal physiology. Based on the above-reported literature, we wanted to prove whether JYTL enhanced PINK1/Parkin-mediated mitophagy through the SIRT1-PGC-1α signaling pathway. The results of Western blotting confirmed that PINK1 and Parkin decreased in the model group, suggesting that PINK1/Parkin-mediated mitophagy was inhibited in SHRs. We also observed a decrease of Beclin1, a key regulator of autophagic flux, and an increase of P62 in SHR models. The elevated p62 levels in SHRs validated poor autophagic clearance, and the above results were reversed by JYTL treatment. Additionally, SIRT1 knockdown by using Ex527 significantly inhibited PINK1/Parkin-mediated mitophagy, suggesting that mitophagy induction may be mediated by SIRT1 activation. To reflect the degree of mitophagy intuitively, we observed the morphological changes of mitochondria and mitophagy in kidney tissues by TEM. We found more mitophagosomes in JYTL-treated rats, but not in the EX527 + JYTL group.

Despite these findings, our research has several limitations. First, JYTL decoction contains a variety of TCM ingredients, it is difficult to determine the most active ingredient(s) in this prescription that interferes with HN. In the future, we are attempted to further identify the key bioactive components in JYTL by using a suite of modern techniques and associated bioactivity assays. In addition, some of the experiments have a low sample size, which potentially limits the statistical robustness and reproducibility and reliability of the results. We will increase the sample size in future studies and apply more rigorous statistical methods such as permutation or bootstrap tests to improve the accuracy, specialty, and rigorousness of the study. Moreover, autophagic activation has different effects in kidneys with different statuses or under different stress factors, the precise mechanism by which JYTL regulates the SIRT1/PGC-1α/mitophagy axis in HN needs further investigation.

## 5 Conclusion

Taken together, this study demonstrated the effects of JYTL decoction on the pathogenesis of HN and elucidated the underlying mechanisms that may be involved in the SIRT1/PGC-1α/mitophagy pathway. We revealed that TLYS decoction can ameliorate TIF and improve renal function in SHRs. This renoprotective effect may be related to improvements in mitochondrial function. The results of our study provide useful information for the treatment of HN using TCM by regulating mitophagy via the SIRT1 pathway.

## Data Availability

The datasets presented in this study can be found in online repositories. The names of the repository/repositories and accession number(s) can be found in the article/Supplementary Material.
