# Supplementary material for: JiangyaTongluo decoction ameliorates tubulointerstitial fibrosis via regulating the SIRT1/PGC-1α/mitophagy axis in hypertensive nephropathy
Source: Front Pharmacol. 2024 Dec 12;15:1491315. doi: 10.3389/fphar.2024.1491315 (PMC11669701; doi:10.3389/fphar.2024.1491315)
Supplement: Supplementary file 1 [file Table2.docx]

**Supplementary Table S2** UPLC-QE/MS data of the identified components in rat serum after oral administration of JYTL decoction.

| NO. | MS | Retention time (min) | Formula | Ion mode | Compounds | Origin |
| --- | --- | --- | --- | --- | --- | --- |
| 1 | 141.0181 | 2.29 | C6H6O3 | [M+H]+ | 5-hydroxymethylfurfural | Achyranthis Bidentatae Radix |
| 2 | 307.0830 | 2.91 | C15H14O6 | [M+H]+ | Epicatechin | Spatholobus Suberectus Dunn |
| 3 | 218.9949 | 4.22 | C7H6O3 | [M+H]+ | 3,4-dihydroxybenzaldehyde | Cassiae Semen,  Radix Salviae |
| 4 | 261.0055 | 13.68 | C9H8O4 | [M+H]+ | Trans caffeic acid | Carthami Flos,  Chrysanthemi Flos |
| 5 | 187.0965 | 16.99 | C9H16O4 | [M+H]+ | Azelaic acid | Achyranthis Bidentatae Radix, |
| 6 | 447.0920 | 19.78 | C21H18O11 | [M+H]+ | Baicalin | Scutellariae Radix |
| 7 | 285.0754 | 19.84 | C16H12O4 | [M+H]+ | Formononetin | Spatholobus Suberectus Dunn |
| 8 | 535.1809 | 20.22 | C27H30O14 | [M+H]+ | Chrysophanol-1-beta-gentiobioside | Cassiae Semen |
| 9 | 267.1588 | 22.22 | C15H22O2 | [M+H]+ | Deca-4,6-diynyl 3-methylbutanoate | Carthami Flos |
| 10 | 323.0909 | 23.93 | C17H12O4 | [M+H]+ | Nortanshinone | Radix Salviae |
| 11 | 271.0597 | 24.30 | C15H10O5 | [M+H]+ | Baicalein | Scutellariae Radix |
| 12 | 391.1723 | 24.73 | C22H26O8 | [M+H]+ | Lirioresinol a | Scutellariae Radix  Carthami Flos |
| 13 | 321.1006 | 25.34 | C19H14NO4+ | [M+H]+ | Coptisine | Achyranthis Bidentatae Radix  Scutellariae Radix |
| 14 | 275.1274 | 25.48 | C16H16O4 | [M+H]+ | (-)-vestitol | Spatholobus Suberectus Dunn |
| 15 | 315.1198 | 25.48 | C18H16O4 | [M+H]+ | Tanshinol b | Radix Salviae |
| 16 | 259.0962 | 25.69 | C16H14O6 | [M+H]+ | 5,7-dihydroxy-2-(3-hydroxy-4-methoxyphenyl)chroman-4-one | Chrysanthemi Flos |
| 17 | 307.0960 | 25.74 | C18H14O3 | [M+H]+ | 1,2-dihydrotanshiquinone | Radix Salviae |
| 18 | 961.4652 | 26.26 | C46H70O19 | [M+H]+ | Achyranthoside e | Achyranthis Bidentatae Radix |
| 19 | 429.1172 | 28.10 | C22H22O10 | [M+H]+ | 1-methoxy-2-[(beta-d-glucopyranosyloxy)methyl]-3-hydroxy-9,10- anthraquinone | Cassiae Semen |
| 20 | 273.1115 | 29.50 | C16H14O4 | [M+H]+ | Alpinetin | Scutellariae Radix |
| 21 | 315.1561 | 30.13 | C19H22O4 | [M+H]+ | Cryptotanshinone | Radix Salviae |
| 22 | 299.1264 | 32.33 | C19H18O6 | [M+H]+ | Tetramethylscutellarein | Radix Salviae |
| 23 | 447.3096 | 32.82 | C26H42O4 | [M+H]+ | Maxacalcitol | Chrysanthemi Flos,  Scutellariae Radix |
| 24 | 365.2871 | 34.33 | C19H38O4 | [M+H]+ | Glycerol palmitate | Carthami Flos |
| 25 | 297.1458 | 36.07 | C19H18O3 | [M+H]+ | Tanshinone iia | Radix Salviae |
| 26 | 271.1686 | 36.26 | C19H22O4 | [M+H]+ | Neocryptotanshinone | Radix Salviae |
| 27 | 356.1537 | 29.37 | C19H21NO5 | [M-H]- | Hmp-hmpep | Achyranthis Bidentatae Radix |
| 28 | 283.1528 | 30.26 | C16H24O6 | [M-H]- | Aarthamoside a2 | Carthami Flos |
| 29 | 327.1238 | 30.90 | C19H18O7 | [M-H]- | Chrysoobtusin | Cassiae Semen |
| 30 | 237.1853 | 32.29 | C15H30O2 | [M-H]- | Pentadecanoic acid | Carthami Flos |
| 31 | 263.2014 | 33.50 | C17H32O2 | [M-H]- | Methyl palmitelaidate | Chrysanthemi Flos，  Scutellariae Radix |
